# Compassionate use of the Seraph® 100 affinity blood filter in pediatric refractory streptococcal septic shock: a case report

**DOI:** 10.3389/fped.2026.1794907

**Published:** 2026-04-17

**Authors:** Raul Montero-Yeboles, Laura Maria Saez-García, Esther Ulloa-Santamaria, Ana Diaz-Vico, Maria Jose Lorenzo-Montero, Beatriz Ruiz-Saez

**Affiliations:** 1 Pediatric Critical Care Unit, Reina Sofia Hospital, Cordoba, Spain; 2 Infectious Diseases Department, Reina Sofia Hospital, Cordoba, Spain

**Keywords:** extracorporeal blood purification, hemoperfusion, pediatric septic shock, Seraph 100, streptococcus pygenes

## Abstract

**Background:**

Septic shock remains a major cause of morbidity and mortality in pediatric intensive care units, particularly in refractory cases unresponsive to standard therapy. Invasive *Streptococcus pyogenes* infection can lead to streptococcal toxic shock syndrome, characterized by severe inflammatory dysregulation and rapid clinical deterioration. Extracorporeal blood purification therapies, such as pathogen-binding hemoperfusion, have emerged as potential adjunctive treatments.

**Case Presentation:**

We report the compassionate use of the Seraph® 100 Microbind® affinity blood filter in a previously healthy 7-year-old girl with refractory septic shock caused by invasive *S. pyogenes*. Despite early initiation of broad-spectrum antibiotics, immunoglobulins, vasopressors, mechanical ventilation, and continuous renal replacement therapy, the patient developed severe multiorgan dysfunction and persistent hemodynamic instability. Hemoperfusion was initiated 42 h after pediatric intensive care unit admission. Due to circuit instability, treatment was performed as a stand-alone 4-h session. Following hemoperfusion, a progressive reduction in vasoactive support and improvement in metabolic parameters, including lactate clearance, were observed within 24–48 h. The patient stabilized without requiring veno-arterial extracorporeal membrane oxygenation. However, early severe peripheral ischemia led to bilateral lower-limb amputations. The patient survived and was discharged after prolonged hospitalization.

**Conclusion:**

In this case, pathogen-binding hemoperfusion with the Seraph® 100 filter was technically feasible and temporally associated with hemodynamic and metabolic improvement in refractory pediatric septic shock. Although causality cannot be established, these findings suggest a potential role for hemoperfusion as an adjunctive rescue therapy in selected cases. Further prospective studies are needed to determine its safety, optimal timing, and clinical efficacy in pediatric populations.

## Background

Sepsis and septic shock remain major causes of morbidity and mortality in pediatric intensive care units (PICUs) worldwide despite advances in antimicrobial therapy, hemodynamic resuscitation, and organ support (1). Mortality is particularly high in refractory septic shock, characterized by persistent circulatory failure, severe metabolic derangement, vasoplegia, and progressive multiorgan dysfunction despite maximal conventional therapy (1).

*Streptococcus pyogenes* (group A streptococcus, GAS) is a frequent paediatric pathogen usually associated with mild infections such as pharyngitis or skin infections (2). However, invasive GAS disease may occasionally lead to fulminant septic shock and streptococcal toxic shock syndrome (3). In these cases, disease severity is driven not only by pathogen burden but also by an exaggerated host immune response mediated by exotoxins, superantigens, cytokine release, endothelial injury, and microcirculatory dysfunction (3).

Increasing understanding of the role of circulating pathogens and pathogen-associated molecules in septic shock pathophysiology has prompted interest in extracorporeal blood purification strategies as potential adjunctive rescue therapies when conventional management fails (4).

The Seraph® 100 Microbind® Affinity Blood Filter is a pathogen-binding hemoperfusion device composed of heparin-coated polyethylene beads designed to mimic endothelial heparan sulfate. Through this mechanism, circulating bacteria, viruses, and pathogen-associated molecules may bind to the device surface and be removed from the bloodstream (5). Experimental studies also suggest that heparin-coated surfaces may interact with circulating cytokines and inflammatory mediators (6).

Available pharmacokinetic data indicate that the device does not significantly remove commonly used antibiotics, allowing antimicrobial therapy to continue during treatment (7, 8).

Clinical experience with the Seraph® 100 filter in pediatric patients remains limited, with only a small number of case reports available, including adolescents and younger pediatric patients with severe viral infections (9–12).

We report the compassionate clinical use of the Seraph® 100 affinity blood filter in a child with refractory septic shock caused by invasive *Streptococcus pyogenes*, focusing on technical feasibility, physiologic response, and clinical outcomes.

## Case presentation

A previously healthy 7-year-old girl (32 kg) was transferred to our tertiary paediatric intensive care unit with rapidly progressive septic shock.

Symptoms began approximately 24 h before admission, initially consisting of vomiting and diarrhea. The patient presented to a regional hospital approximately three hours before transfer, where progressive hypotension, tachycardia, and prolonged capillary refill were observed, prompting urgent transfer to our tertiary center. The major clinical events during the patient's hospitalization are summarized in Table 1.

**Table 1:** Chronological timeline of major clinical events.

Event	Time relative to PICU admission
Symptom onset	−24 h
Admission to regional hospital	−3 h
Transfer to tertiary center	−1 to 0 h
PICU admission	0 h
Initiation of antibiotic therapy	0 h
Intubation and mechanical ventilation	+2 h
Initiation of corticosteroid therapy	+2 h
Intravenous immunoglobulin (IVIG) administration	+6 h
Initiation of continuous renal replacement therapy (CRRT)	+7 h
Initiation of Seraph® 100 hemoperfusion	+42 h
Completion of Seraph® hemoperfusion	+46 h

Timeline of major diagnostic and therapeutic interventions from symptom onset to hemoperfusion therapy. Time 0 corresponds to admission to the pediatric intensive care unit (PICU).

At PICU admission the patient presented with severe circulatory shock and multiorgan dysfunction. Laboratory evaluation demonstrated marked leukocytosis (18,730/µL), severe metabolic acidosis (pH 6.99), hyperlactatemia (15.3 mmol/L), acute kidney injury (creatinine 4.98 mg/dL), thrombocytopenia, and coagulopathy (INR 2.02). Peripheral ischemic lesions affecting the distal extremities were already present at admission.

Blood cultures and rapid antigen testing confirmed infection with Streptococcus pyogenes. These findings supported the diagnosis of invasive *Streptococcus pyogenes* infection with streptococcal toxic shock syndrome. Broad-spectrum antimicrobial therapy with cefotaxime and clindamycin was initiated immediately, together with intravenous immunoglobulin, according to recommendations for severe invasive streptococcal infections.

Despite aggressive resuscitation following international pediatric sepsis guidelines, the patient developed refractory septic shock with escalating vasopressor requirements, severe metabolic derangement, and progressive vasoplegia.

Endotracheal intubation was required two hours after PICU admission, and continuous renal replacement therapy was initiated seven hours after admission due to persistent anuria and severe metabolic acidosis.

The clinical severity and evolution of key laboratory parameters are summarized in Table 2, which illustrates the extent of organ dysfunction and inflammatory response during the course of illness.

**Table 2:** Laboratory parameters during the clinical course.

Parameter	PICU admission	Pre-Seraph	Post-Seraph	24 h after Seraph	PICU discharge
Inflammatory markers					
C-reactive protein (CRP), mg/L	221	240	251	264.6	40.2
Procalcitonin (PCT), ng/mL	>50	>50	>50	>50	0.21
Ferritin, ng/mL	–	–	–	–	–
Interleukin−6 (IL−6), pg/mL	–	–	–	–	–
Metabolic parameters					
Lactate, mmol/L	15.3	12.8	13.4	12.4	1.4
pH	6.99	7.38	7.36	7.41	7.42
Base excess, mmol/L	−23.2	1	−0.5	2.7	−0.4
Bicarbonate, mmol/L	8.2	26	24.9	27.3	24
Anion gap	25.1	14.4	16.1	12.9	13.1
Organ function					
Creatinine, mg/dL	4.98	2	1.17	0.92	2.08
Urea, mg/dL	82	45	31	28	54
AST/ALT, U/L	245/100	603/186	496/172	449/178	37/40
Total bilirubin, mg/dL	1.7	3.6	3.1	3.2	0.7
INR	2.02	2.31	1.46	1.13	1
aPTT ratio	1.6	2.4	2.4	2	1.1
Fibrinogen, mg/dL	250	157	125	134	250
D-dimer	–	–	–	–	–
Hematology					
Hemoglobin, g/dL	13.1	12.8	11.4	10.3	7.3
Leukocytes,/µL	18,730	5,700	14,820	21,770	18,660
Neutrophils,/µL	17,900	4,640	12,370	17,630	14,600
Lymphocytes,/µL	1,500	400	480	1,520	2,570
Platelets,/µL	149,000	22,000	19,000	20,000	689,000
Proteins					
Total protein, g/dL	5.1	4.7	3.9	4.0	7.3

Laboratory values from PICU admission through hemoperfusion therapy with the Seraph® 100 affinity blood filter. ‘Pre-Seraph’ represents the last measurement obtained before initiation of hemoperfusion, while ‘Post-Seraph’ corresponds to the first measurement obtained after completion of the treatment session. PICU, pediatric intensive care unit; CRP, C-reactive protein; PCT, procalcitonin; AST, aspartate aminotransferase; ALT, alanine aminotransferase; INR, international normalized ratio; aPTT, activated partial thromboplastin time.

### Extracorporeal therapy

Given ongoing deterioration despite maximal supportive therapy and consideration of veno-arterial extracorporeal membrane oxygenation (VA-ECMO) as a rescue therapy, pathogen-binding hemoperfusion with the Seraph® 100 filter was initiated 42 h after PICU admission following parental consent.

In the European clinical context, the use of this device was performed as compassionate clinical use of an approved medical device outside its standard indication, according to institutional regulations.

Hemoperfusion was initially attempted in-line with the CRRT circuit. However, clotting and circuit instability occurred, likely related to the combination of severe sepsis-associated coagulopathy, high inflammatory burden, and the additional extracorporeal resistance introduced by the hemoperfusion cartridge. For this reason, the therapy was subsequently performed as a stand-alone hemoperfusion session, temporarily disconnecting the patient from CRRT during treatment.

The device was primed with 1 L of 0.9% saline solution, and hemoperfusion was performed for 4 h using systemic anticoagulation with low-dose unfractionated heparin (5 IU/kg/h) due to the underlying coagulopathy.

Technical parameters of the extracorporeal therapies used during the patient's clinical course are summarized in Table 3.

**Table 3:** Extracorporeal therapy parameters.

Parameter	Value
Patient characteristics	
Body weight	32 kg
Body surface area	1.07 m^2^
Estimated blood volume	1,344 mL
Hemodynamic monitoring	
Monitoring methods	Invasive arterial blood pressure, central venous pressure, pulse oximetry
Vascular access	
Medication access	7 Fr triple-lumen catheter, 30 cm, femoral vein
CRRT access	11 Fr catheter, 15 cm, internal jugular vein
Continuous renal replacement therapy (CRRT)	
Modality	Continuous veno-venous hemodiafiltration (CVVHDF)
Dialysate dose	1.5 L/1.73 m^2^/h
Replacement dose	40 mL/kg/h
Ultrafiltration target	50–100 mL/h after stabilization
CRRT anticoagulation	Unfractionated heparin
Heparin dose	5 IU/kg/h
Seraph® 100 hemoperfusion	
Indication	Refractory septic shock
Timing of initiation	42 h after PICU admission
Number of sessions	1
Treatment duration	4 h
Priming solution	1 L of 0.9% saline
Anticoagulation	Low-dose unfractionated heparin
Target aPTT	1–1.2
CRRT during hemoperfusion	Temporarily interrupted due to circuit instability
Circuit configuration	Standalone hemoperfusion session

Technical characteristics of extracorporeal therapies used during the patient's clinical course, including continuous renal replacement therapy (CRRT) and pathogen-binding hemoperfusion with the Seraph® 100 affinity blood filter.

The decision to perform a single 4-hour session was based on several factors, including hemodynamic instability, circuit clotting risk, and the intention to evaluate early physiologic response before considering prolonged treatment.

## Outcome

Following completion of hemoperfusion, a progressive reduction in vasoactive support was observed within the subsequent 12–24 h, accompanied by gradual metabolic stabilization and decreasing lactate levels.

**Figure 1:**
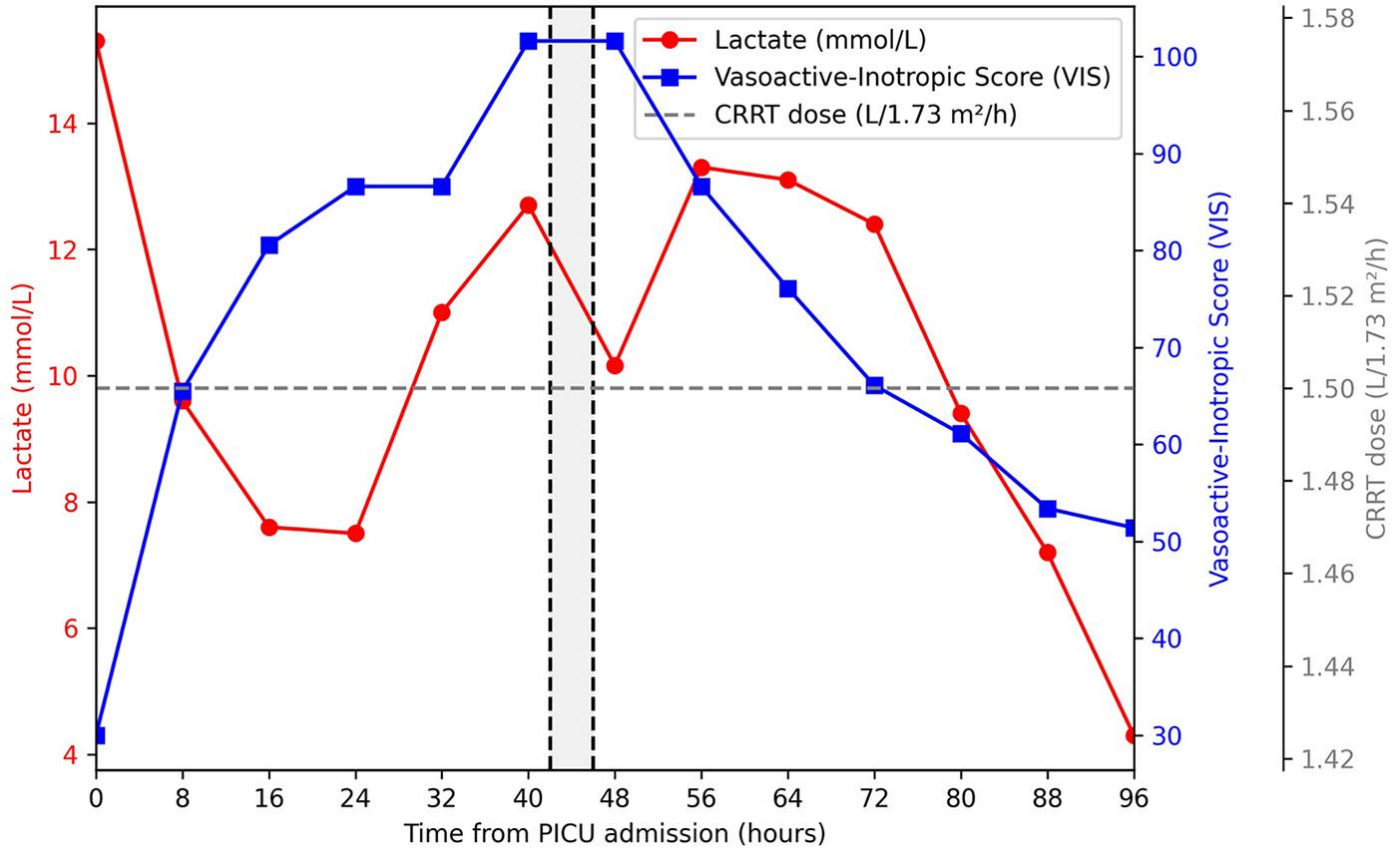
Evolution of lactate levels and vasoactive support during the early course of septic shock. Temporal evolution of serum lactate concentrations (red line) and vasoactive-inotropic score (VIS) (blue line) during the first 96 h following PICU admission. Vertical dashed lines indicate the start and end of hemoperfusion therapy with the Seraph® 100 affinity blood filter (42–46 h after PICU admission). Continuous renal replacement therapy (CRRT) dose remained stable during this period (gray dashed line). Following hemoperfusion therapy, a progressive decline in vasoactive requirements and subsequent normalization of lactate levels was observed.

During the first 24 h following hemoperfusion, the vasoactive-inotropic score decreased progressively while lactate levels subsequently normalized over the following days, as illustrated in Figure 1. The patient ultimately stabilized sufficiently to avoid escalation to veno-arterial extracorporeal membrane oxygenation (VA-ECMO) support.

However, peripheral ischemia progressed during the early phase of shock and ultimately required bilateral lower-limb amputations. Ischemic lesions had been present since PICU admission and were likely related to a combination of streptococcal toxic shock, disseminated intravascular coagulation, microvascular thrombosis, and prolonged exposure to high-dose vasopressors.

The patient survived the septic episode and was discharged from the PICU after approximately two months of hospitalization.

## Discussion

The pathophysiology of septic shock involves both pathogen burden and dysregulated host immune responses, including excessive inflammatory activation and endothelial injury (13, 14). In invasive *Streptococcus pyogenes* infections, exotoxins and superantigens can trigger massive immune activation and microcirculatory collapse (3). These virulence factors may amplify systemic inflammation through superantigen-mediated T-cell activation and cytokine release, potentially contributing to the profound vasoplegia observed in streptococcal toxic shock.

The Seraph® 100 device is designed to remove circulating pathogens through heparin-mimetic binding mechanisms that mimic endothelial heparan sulfate interactions (5). Experimental data suggest that heparin-coated surfaces may also interact with circulating cytokines and inflammatory mediators (6).

In addition to direct pathogen removal, it has been hypothesized that reduction of circulating pathogen load may allow partial restoration of immune homeostasis, enabling the host immune system to regain regulatory balance and potentially attenuate the dysregulated inflammatory response characteristic of septic shock (13).

In this case, hemoperfusion therapy was temporally associated with rapid lactate decline and progressive reduction in vasoactive support. Importantly, this improvement occurred without changes in CRRT settings, suggesting that metabolic stabilization was unlikely to be explained solely by renal replacement therapy.

Although causality cannot be established from a single observational case, the temporal association between hemoperfusion therapy and the subsequent reduction in vasoactive support, followed by normalization of lactate levels, may suggest a potential physiologic contribution of pathogen-binding hemoperfusion in the setting of fulminant septic shock.

## Limitations

This case report has several important limitations that restrict the interpretation of the observed clinical course.

First, the report describes a single patient, which inherently limits the ability to draw causal conclusions regarding the therapeutic effect of hemoperfusion.

Second, cytokine concentrations, endotoxin levels, and antibiotic plasma concentrations were not measured during treatment because these laboratory tests were not available at the time the therapy was initiated. The absence of these data prevents mechanistic assessment of pathogen removal or immunomodulatory effects associated with the device.

Third, although several clinical and laboratory parameters were recorded, not all extracorporeal circuit parameters could be retrospectively reconstructed with complete precision, which limits the ability to fully evaluate technical aspects of the treatment.

Finally, the patient received multiple simultaneous therapies including antimicrobial treatment, vasopressors, immunoglobulin administration, corticosteroids, and renal replacement therapy. Therefore, the observed clinical improvement cannot be attributed solely to hemoperfusion. However, it is noteworthy that these supportive therapies had been in place for nearly two days prior to the initiation of hemoperfusion without clear clinical benefit, during which time the patient continued to show progressive hemodynamic and metabolic deterioration. The initial signs of stabilization, including a gradual reduction in vasoactive requirements followed by improvement in lactate levels, were observed only after the hemoperfusion session. Although causal inference cannot be established from a single case, the temporal relationship between hemoperfusion and subsequent clinical improvement should be interpreted cautiously but may be considered hypothesis-generating.

## Conclusion

The compassionate clinical use of the Seraph® 100 affinity blood filter was technically feasible in this critically ill child with refractory septic shock caused by *Streptococcus pyogenes*.

Hemoperfusion was temporally associated with a progressive reduction in vasoactive support and subsequent metabolic stabilization, occurring in a patient who had previously demonstrated clinical deterioration despite maximal conventional therapy and was being considered for escalation to extracorporeal life support.

Although causality cannot be established from a single observational case, this report highlights the potential role of pathogen-binding hemoperfusion as an adjunctive rescue therapy in selected cases of fulminant pediatric septic shock.

Further prospective studies are required to better define the safety, optimal timing, and potential clinical benefit of Seraph® 100 hemoperfusion in pediatric patients with severe infections.

## Data Availability

The datasets presented in this article are not publicly available due to patient privacy and institutional data protection regulations. Requests to access anonymized data should be directed to the corresponding author and will be considered in accordance with institutional policies.
